# Supplementary material for: MetaRibo-Seq measures translation in microbiomes
Source: Nat Commun. 2020 Jun 29;11:3268. doi: 10.1038/s41467-020-17081-z (PMC7324362; doi:10.1038/s41467-020-17081-z)
Supplement: Supplementary file 10 — Supplementary Data 7 [file 41467_2020_17081_MOESM10_ESM.zip › File2/Confidence_High_Taxonomy/291374_out.krona.html]

Javascript must be enabled to view this page.

members
magnitude
magnitudeUnassigned
count
unassigned
taxon
rank

291374\_out

90

superkingdom
90
2

976
phylum
90

90
class
200643

171549
90
order

1
family
2005525

375288
1
genus


SRS098073\_contig\_number\_1085
species
1
46503

88
family
815

30
816
88

SRS012849\_contig\_number\_29492SRS013098\_contig\_number\_contig-100\_3466.294640SRS013476\_contig\_number\_36290SRS016203\_contig\_number\_contig-100\_8905.48345SRS016381\_contig\_number\_contig-100\_532.533SRS017701\_contig\_number\_contig-100\_5686.117412SRS018351\_contig\_number\_16646SRS024132\_contig\_number\_19404SRS049712\_contig\_number\_contig-100\_6756.124198SRS049900\_contig\_number\_15621SRS050299\_contig\_number\_11326SRS050974\_contig\_number\_contig-100\_3271.3272SRS051882\_contig\_number\_contig-100\_10020.103240SRS053649\_contig\_number\_contig-100\_12841.109198SRS056273\_contig\_number\_17442SRS056519\_contig\_number\_5334SRS075078\_contig\_number\_13444SRS077454\_contig\_number\_14724SRS078242\_contig\_number\_14291SRS078260\_contig\_number\_contig-100\_1134.1134SRS078419\_contig\_number\_34309SRS097958\_contig\_number\_contig-100\_1433.136674SRS104636\_contig\_number\_26040SRS1055083\_contig\_number\_11795SRS143722\_contig\_number\_12643SRS147039\_contig\_number\_2016SRS147088\_contig\_number\_959SRS147346\_contig\_number\_43724SRS148196\_contig\_number\_13492SRS893383\_contig\_number\_contig-100\_4670.190969
genus

species
2

SRS064645\_contig\_number\_390SRS893270\_contig\_number\_16182
338188

1263038

SRS893342\_contig\_number\_1437
1
species

species
1

SRS043768\_contig\_number\_23816
47678

820
34
species

SRS012273\_contig\_number\_46006SRS013638\_contig\_number\_11829SRS014235\_contig\_number\_7838SRS015264\_contig\_number\_11104SRS015694\_contig\_number\_17652SRS018575\_contig\_number\_8087SRS018836\_contig\_number\_14600SRS019445\_contig\_number\_16266SRS019685\_contig\_number\_13474SRS020328\_contig\_number\_39504SRS020394\_contig\_number\_14643SRS022071\_contig\_number\_34703SRS042628\_contig\_number\_33192SRS046068\_contig\_number\_119SRS049446\_contig\_number\_10098SRS063928\_contig\_number\_189SRS074586\_contig\_number\_4657SRS075773\_contig\_number\_32493SRS077589\_contig\_number\_4401SRS098717\_contig\_number\_8509SRS100021\_contig\_number\_973SRS1041032\_contig\_number\_11521SRS1041112\_contig\_number\_2490SRS1041129\_contig\_number\_3629SRS1041157\_contig\_number\_7154SRS104400\_contig\_number\_27092SRS104975\_contig\_number\_13114SRS144135\_contig\_number\_15964SRS144297\_contig\_number\_2547SRS144714\_contig\_number\_29160SRS146812\_contig\_number\_57720SRS148091\_contig\_number\_12704SRS149325\_contig\_number\_11409SRS971276\_contig\_number\_17756

1
species

SRS050520\_contig\_number\_175
2292926

357276
2
species

SRS019286\_contig\_number\_3586SRS075021\_contig\_number\_3009

817

SRS142599\_contig\_number\_14941
1
species

2
species

SRS016018\_contig\_number\_10480SRS098655\_contig\_number\_1999
28116


SRS016989\_contig\_number\_13731
species
1
477666


SRS063518\_contig\_number\_31543SRS077086\_contig\_number\_3572SRS1041164\_contig\_number\_2255SRS143372\_contig\_number\_7210SRS147977\_contig\_number\_3594
species
5
1946089


SRS143876\_contig\_number\_1202
species
1
585543

2292928
1
species

SRS1041130\_contig\_number\_3184

310300

SRS1054716\_contig\_number\_10203
1
species

371601
species
1

SRS048981\_contig\_number\_4203

2292931

SRS1041145\_contig\_number\_40150
species
1

821

SRS149075\_contig\_number\_11015
1
species

626930

SRS149181\_contig\_number\_30114
species
1

674529

SRS015579\_contig\_number\_14156
1
species

171550
1
family

1
genus
239759

28117

SRS098061\_contig\_number\_11805
1
species
